# Recycled Polystyrene as a Sustainable Material for Hollow Fiber Membranes in Dye Filtration

**DOI:** 10.3390/membranes15100285

**Published:** 2025-09-23

**Authors:** Mauricio Huhn-Ibarra, Libia Madai Itza-Uitzil, Marcial Yam-Cervantes, Abigail González-Díaz, Fernando José Zapata-Catzin, Javier Ivan Cauich-Cupul, Manuel Aguilar-Vega, Maria Ortencia González-Díaz

**Affiliations:** 1 Centro de Investigación Científica de Yucatán, A.C., Calle 43 No. 130, Chuburná de Hidalgo, Mérida 97200, Yucatán, Mexico; huhn1234@hotmail.com (M.H.-I.);; 2 Tecnológico Nacional de México, Instituto Tecnológico de Mérida, Av. Tecnológico S/N Km. 4.5, Mérida 97118, Yucatán, Mexico; 3 El Colegio de Puebla A.C., Av. 141 pte. 505, Gabriel Pastor 1ra. Sección, Heroica Puebla de Zaragoza 72420, Puebla, Mexico; 4 SECIHTI-Centro de Investigación Científica de Yucatán, A.C., Calle 43 No. 130, Chuburná de Hidalgo, Mérida 97200, Yucatán, Mexico

**Keywords:** hollow fiber membranes, recycling expanded polystyrene, polymer blend, dye rejection, water treatment

## Abstract

Expanded polystyrene (EPS) waste was chemically modified by sulfonation to obtain sulfonated EPS (sEPS), which was subsequently blended with virgin polyphenylsulfone (PPSU) at concentrations ranging from 10 to 50% to elaborate hollow fiber membranes for dye removal. The membranes were elaborated by non-solvent-induced phase separation and characterized by scanning electron microscopy, mechanical properties, antifouling, water flux measurements, and dye rejection performance. Scanning electron microscopy images of PPSU/sEPS blends showed well-defined membrane cross-sections with no polymer segregation up to 30% recycled EPS content, indicating improved compatibility due to EPS sulfonation. The HFMs present mean pore radii ranging from 4.2 ± 0.5 to 11.1 ± 1.0 nm with porosity up to 80%. Water flux improved significantly from 3.1 to 21.2 L m^−2^ h^−1^ at 2 bar as sEPS content increased. Dye rejection performance was promising, with Reactive Black 5 rejection ranging from 77% to 99%. The 80/20s PPSU/sEPS membrane showed the highest Reactive Black 5 rejection at 98.3% and revealed a 70.3% rejection in a 24 h dye mixture test. Furthermore, the 70/30s displayed superior anti-fouling properties, achieving a 99.3% flux recovery ratio in a xanthan gum solution at 2 bar. This study demonstrates a novel approach to transform EPS waste into high-performance hollow fiber membrane with competitive antifouling and dye separation properties.

## 1. Introduction

The progressive increase in plastic production and the cumulative production of polymers since 1950, combined with the fact that less than 10% of plastic waste is recycled, mean that we may soon coexist with around 26 billion tons of plastic waste [1]. In this context, increasing plastic waste recycling is crucial for environmental protection and promoting a circular economy. However, single-use plastics present a significant challenge for waste management and recycling efforts [2]. EPS is a single-use plastic employed in various applications, such as disposable dishes, packaging materials, laboratory wares, and food storage containers, among others. Its consumption has risen not only due to its ease of use and low cost but also due to the rapid growth in the global population, which drives higher demand and waste generation. EPS poses significant environmental and health risks to both wildlife and humans. Due to its low density, it is easily dispersed by wind, contributing to plastic pollution in various ecosystems, even reaching remote areas where it has no place. As a non-biodegradable material, EPS persists in the environment for thousands of years, gradually breaking down into small particles that are often mistaken for food by wildlife [3]. Moreover, its recycling is difficult due to its lightweight, low-density structure and the large volume, which, combined with the inefficiency of chemical recycling, results in high processing costs. As a consequence, the lack of adequate waste management infrastructure often leads to open burning, releasing toxic gases that contribute to climate change and emit carcinogens [4].

Recently, single-use EPS waste has gained attention as a promising material for the circular economy. For example, ion-exchange resins have been developed from sulfonated EPS waste for heavy metals removal and for norfloxacin adsorption [5,6]. Sulfonation introduces sulfonic acid (–SO_3_H) groups onto the aromatic pendant units of EPS, significantly enhancing its hydrophilicity and enabling ionic interactions. In addition, the presence of negatively charged sulfonic groups introduces electrostatic interactions with cationic solutes, which is particularly advantageous for dye adsorption and membrane-based separation processes [5]. Despite these advantages, the application of sulfonated EPS in membrane fabrication remains limited, with few reports exploring its application beyond separation systems. Moreover, flat-sheet membranes based on unmodified EPS waste have been successfully fabricated by the phase inversion method or electrospinning for water treatment [7,8,9,10,11,12,13]. However, the development of EPS-based hollow fiber membranes (HFMs) has not yet been reported. HFMs are widely used in water treatment processes, especially for ultrafiltration (UF) and nanofiltration (NF), due to their high surface area, flexibility, and efficiency [14]. Although EPS waste presents good HFM-forming ability, the resulting membranes are brittle and difficult to handle since they cannot withstand pressure. To address these shortfalls, recycled polymers are often blended with virgin polymers to achieve the desired mechanical properties [15]. For example, flat asymmetric membranes composed of different mass percentages (%) of polysulfone (PSU) as the basic matrix, with recycled EPS as an additive, and N,N′-dimethyl formamide as a solvent, have been reported [16]. The authors found that the incorporation of a small amount of EPS (3%) into the PSU matrix enhanced both the mechanical strength and separation performance of the resulting membrane, achieving high water permeation values on the order of 2970 L m^−2^.bar^−1^. Moreover, HFMs were elaborated from poly(vinyl chloride)/polystyrene (16/1 wt% PVC/PS) blends with PEG as an additive and N,N′-dimethylacetamide as the solvent. The addition of poly(ethylene glycol) (PEG) significantly influenced membrane morphology, pore size, and water permeability [17]. Likewise, low-cost ultrafiltration membranes based on waste EPS blended with polyvinylidene fluoride (PVDF) were prepared by phase inversion. Increasing EPS content changed membrane morphology and improved dye rejection, reaching up to 81% for Congo red at 50% EPS [18]. In general, blending helps to reduce costs, while the new blended fiber properties can still deliver outstanding performance [19].

Nevertheless, polymer pairs are often immiscible, leading to heterogeneous morphologies as a result of phase separation. Phase separation causes the formation of large, discrete domains within the continuous matrix, which can significantly affect the material’s performance, particularly its mechanical properties. One effective strategy to improve the compatibility of immiscible polymer blends is to modify the chemical composition of one or both components by introducing functional groups that can interact favorably with the other phase [20]. This approach helps to enhance the interfacial adhesion between the phases, leading to a more homogeneous morphology and, consequently, improved material properties.

In this study, a polymer blending approach is proposed to overcome the limitations of EPS waste for HFM formation for dye removal from wastewater. HFMs have demonstrated considerable potential in the separation and removal of synthetic dyes from industrial effluents, particularly persistent azo dyes such as Reactive Black 5. Widely used in textile manufacturing, this dye exhibits high chemical stability and low biodegradability, presenting significant challenges for conventional wastewater treatment methods. By combining PPSU with different amounts of sulfonated EPS waste (sEPS) obtained from packaging materials, HFMs for water treatment and dye removal were prepared. PPSU was chosen over other polyarylsulfones for its higher impact strength, which could significantly improve EPS brittleness, enhancing HFM mechanical properties. HFMs from PPSU/sEPS offer several advantages. First, sulfonating EPS waste enhances its compatibility with PPSU. Second, incorporation of sulfonic acid groups into EPS not only introduces a better interaction for polymer compatibilization but also imparts anti-fouling properties. Furthermore, an optimal PPSU/sulfonated EPS waste blend ratio is determined, as well as the extent to which recycled sulfonated EPS can substitute virgin polymer without compromising performance. This study includes a detailed physical and mechanical characterization of the obtained PPSU/sEPS waste HFMs. Additionally, the separation of Reactive Black 5 and a mixture of four dyes, as well as the antifouling properties of HFMs, was analyzed.

## 2. Materials and Methods

### 2.1. Materials

EPS (77,000 g/mol, measured by Gel Permeation Chromatography, GPC) was obtained from electronic packaging waste. N-Methyl-2-pyrrolidinone anhydrous (NMP, 99.5%), 1-2-dichloroethane (DCE, 99.8%), potassium phosphate di-basic (K_2_HPO_4_, 98%), trimethylsilyl chlorosulfonate (TMSClS, 99%), bovine serum albumin (BSA, 67 kDa), and phenolphthalein (ACS reagent) were used as received from Sigma Aldrich (Toluca, México). Potassium phosphate monobasic (KH_2_PO_4_ 99.5%, Fluka chemical, GmbH, Buchs, Switzerland), ethanol (EtOH, 96% CTR Scientific, Monterrey, México), glycerol (local provider, Yucatán, México), PPSU Radel R-5000 (55,000 g mol^−1^), xanthan gum (XG), Methylene Blue (MB), Black Reactive 5 (BR5), Methyl Orange (MO), and Congo Red (CR) were also used as received.

### 2.2. Polystyrene Sulfonation

EPS waste was dissolved in ethyl acetate and precipitated in ethanol to remove additives. EPS sulfonation was carried out by direct sulfonation with a molar ratio of EPS/ TMSClS of 1:0.05 in a similar manner as the method reported by Sawitri et al. [21]. A 12% (*w*/*v*) EPS in DCE solution was mechanically stirred in a 250 mL three-neck flask until complete dissolution. After that, TMSCIS, diluted to 10% (*v*/*v*) in DCE, was dropwise added to the homogeneous solution. The mixture was then stirred for 6 h at room temperature. The crude product was precipitated in EtOH, filtered, and washed with EtOH. Finally, sulfonated EPS was dried at 70 °C under vacuum for 24 h.

### 2.3. PPSU/sEPS Membrane Elaboration

The HFMs were fabricated by the dry–wet phase inversion process, employing a custom-designed spinneret [22]. An 18% (*w*/*w*) polymeric dope solution was prepared by dissolving the required amounts of PPSU, EPS, and sEPS (see Table 1) in NMP (22.1 mL) at 60 °C for 18 h. The selected blend ratios of PPSU/sEPS were 90/10s, 80/20, 80/20s, 70/30s, 60/40s, and 50/50s (% *w*/*w*), where “s” denotes sulfonated EPS. The external and internal bore HFM diameters fall between 1137.5 ± 138 µm and 570 ± 138 µm, respectively.

The bore fluid and the dope solution were delivered through separate channels of the spinneret with their flow rates independently controlled by separate pumps. Upon exiting the spinneret, the two fluids converged at the tip to form the fiber. Then, the nascent fiber passed through a 3.5 cm air gap before entering a 90-L water coagulation bath. Inside the bath, the fibers settled by gravity at the bottom of the vessel without the aid of take-up rollers. After precipitation, the fibers were collected and rinsed in tap water for further use. A summary of the spinning conditions process is provided in Table 2.

The PPSU/sEPS membranes were treated with a 20 wt% glycerol solution for 24 h prior to module fabrication to prevent the collapse of their porous structure.

### 2.4. Characterization

#### 2.4.1. Characterization of Sulfonated EPS

The sulfonation degree (SD) of EPS was determined by the titration method [23]. In this study, 50 mg of sEPS was dissolved in 10 mL of a methanol/toluene (10/90 *v*/*v*) and titrated with a 0.01 M methanolic NaOH solution. The SD value (%) was calculated using Equation (1):

(1) $$SD\;=\;\frac{104\;\;C_{NaOH}\times V_{NaOH}}{W\;-\;(81\times C_{NaOH}\times V_{NaOH})}$$

where $C_{NaOH}\;$and $V_{NaOH}$ are the concentration (mol/L) and volume (mL) of NaOH/methanol solution, respectively; 81 and 104 are the sulfonic (–SO_3_H) groups and styrene unit molar mass, respectively; and W is the sulfonated EPS weight (g).

EPS waste molecular weight measurements (*Mn* and *Mw*) were carried out by GPC on an Agilent 1100 HPLC system (Agilent Technologies, Santa Clara, CA, USA). The mobile phase was dimethyl formamide (DMF) containing 0.5% lithium bromide (LiBr), with a flow rate of 1.0 mL/min at 30 °C. Polystyrene standards were used for the calibration curve. ^1^H NMR spectra were acquired using a Varian 600 MHz spectrometer (Varian Inc., Palo Alto, CA, USA). The EPS sample was dissolved in CDCl_3_, while sEPS samples were prepared in DMSO-d_6_. FTIR spectra of the samples were recorded using a Nicolet 8700 spectrometer (Thermo Fisher Scientific, Waltham, MA, USA) equipped with an attenuated total reflectance (ATR) accessory, over a wavenumber range of 4000–650 cm^−1^. Differential scanning calorimetry (DSC) measurements were performed using a DSC 7 calorimeter (PerkinElmer, Waltham, MA, USA). Membrane samples were heated from 40 °C to 200 °C at a heating rate of 10 °C/min under a nitrogen atmosphere. Water uptake and weight loss were gravimetrically determined in duplicate as described in the literature [24].

#### 2.4.2. Solubility Parameter Difference and Polymer–Polymer Miscibility

For each polymer, Hansen solubility parameters (HSPs) were estimated using the method described by Van Krevelen, based on group contribution. The dispersive ($\delta_{d}$), polar ($\delta_{p}$), and hydrogen bonding ($\delta_{h}$) interactions were calculated using the following equations [25]:

(2) $$\delta_{d}=\frac{\sum F_{di}}{V_{gi}}$$

(3) $$\delta_{p}=\frac{\sqrt{\sum F_{pi}^{2}}}{V_{gi}}$$

(4) $$\delta_{h}=\sqrt{\frac{\sum E_{hi}}{V_{gi}}}$$

where *V_gi_* is the molar volume and *E_hi_*, *F_di_*, and *F_pi_* are the hydrogen bonding force, dispersion force, and dipole force components of the solubility parameter, respectively.

Polymer–polymer miscibility was calculated using the volume-dependent solubility parameter (*δ_v_*), based on a 2D approach introduced by Bradely. In this method, *δ_p_* and *δ_d_* are used to predict the combined thermodynamic effects on polymer–polymer miscibility, particularly in relation to hydrogen bonding (Equation (5)) [26,27].

(5) $$\delta_{v}=\sqrt{\delta_{d}^{2}+\delta_{p}^{2}}$$

Then, the relative distance in the blend, denoted as (*R_a_*_(*v*)_), is calculated using the Pythagorean Theorem (Equation (6)).

(6) $$R_{a(v)}=\sqrt{{\left(\delta_{v2}-\delta_{v1}\right)}^{2}+{\left(\delta_{h2}-\delta_{h1}\right)}^{2}}$$

A parallel plate rheometer (TA Instruments 2000, model AR-2000, New Castle, DE, USA) with a 20 mm Peltier plate (cone–plate) geometry was used to evaluate the viscosity of the polymer dope solutions. The measurements were taken at a 13.2 s^−1^ constant shear rate.

#### 2.4.3. Membrane Morphology and Mechanical Properties

The cross-sectional morphology of the HFMs was examined using a scanning electron microscope (SEM, JSM-630LV, JEOL Ltd., Tokyo, Japan) at magnifications of ×70 and ×300. The membrane samples were fractured by freeze-fracturing using liquid nitrogen prior to cross-sectional SEM analysis. Before SEM imaging, membrane samples were sputtered with a thin gold layer using a Denton Vacuum Desk II (Moorestown, NJ, USA) sputter coater to enhance surface conductivity. The tensile properties of the HFMs were evaluated using a universal testing machine (AGS-X, Shimadzu Corporation, Kyoto, Japan) equipped with a 100 N load cell. The tests were performed at a crosshead speed of 1 mm/min. Each sample was tested ten times to ensure reproducibility.

#### 2.4.4. Pore Size Radius, Porosity, Water Contact Angle, and Membrane Separation Performance

**Figure 1:**
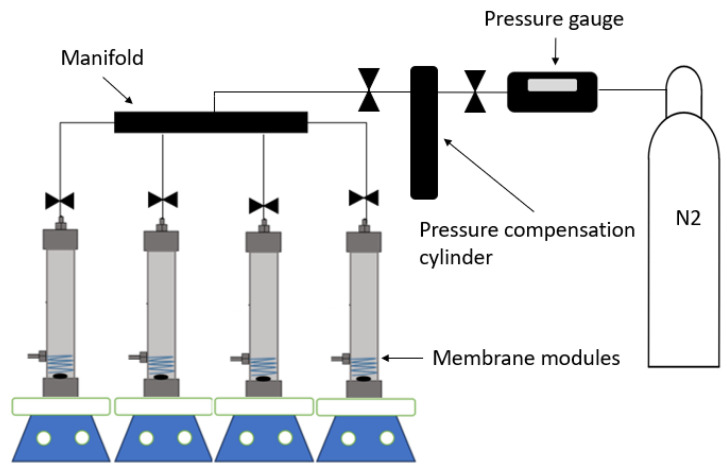
Schematic diagram of the filtration system.

PPSU/sEPS HFMs separation performance was evaluated in a self-developed testing system that simultaneously tests four modules using a custom stirred dead-end configuration (see Figure 1). The modules were fabricated from stainless steel, with dimensions of 30 cm in length and 3.17 cm in diameter, with a 180 mL overall capacity. Inside the modules, two HFMs were placed and wrapped at the bottom, near the magnetic stirrer. The fibers were sealed into modules using epoxy resin. Each module had an effective area of ~1.98 × 10^−3^ m^2^.

Antifouling performance testing: In preparation for the antifouling performance test, HFMs were compacted by passing pure water through the membranes at 2 bar for 1 h. After that, pure water was filtered through the membranes for 30 min to determine the initial flux (*J*_0_), followed by a permeation flux of a 1.0 mg/mL BSA/phosphate-buffered solution or a 1.0 mg/mL XG solution. The BSA and XG solutions permeation was stopped after 1 h, and the membranes were washed by shaking the module with water for 3 min to remove loose proteins attached to them. In the next step, pure water flux (*J*_1_) was again measured for 30 min to complete the antifouling test. During all experiments, the pressure was set at 2 bar or at 3 bar.

The water flux (*J*) in L m^−2^ h^−1^ was calculated using Equation (7).

(7) $$J=\frac{V\;}{A\times t}$$

where *V* is the filtrated volume (L), *A* is the membrane area (m^2^), and *t* is the operation time (h).

For the evaluation of the BSA and XG antifouling test, the flux recovery ratio (FRR) was calculated using Equation (8) [28].

(8) $$FRR\;\left(\%\right)=\left(\frac{J_{1}}{J_{0}}\right)\times 100$$

where *J*_0_ (L m^−2^ h^−1^) is the pure water flux at the beginning, and *J*_1_ (L m^−2^ h^−1^) is the value of pure water flux after membrane cleaning.

Dye rejection experiment: RB5 (50 ppm) and dye mixture solutions were used for dye rejection measurement in each module. For the 50 ppm dye mixture tests, MO, MB, RB5, and CR were used. A UV–vis spectrometer set at 595 nm was used to measure RB5 concentration. For the dye mixture rejection test, a scan between 300 nm and 700 nm was performed before and after the test to determine the wavelength of the highest absorption peak, which was at 485 nm. Rejection percentages were calculated with Equation (9).

(9) $$R\;\left(\%\right)=\left(1-\frac{C_{p}}{C_{f}}\right)\times 100$$

where *C_p_* and *C_f_* are the concentrations of permeate and feed solutions, respectively.

Porosity Measurement: Hollow fiber porosity, ε, was determined by weighing wet and dry methods from HFM according to Equation (10) [29].

(10) $$\varepsilon =\frac{\frac{\left(W_{1}-W_{2}\right)}{\rho_{w}}}{\frac{\left(W_{1}-W_{2}\right)}{\rho_{w}}+\frac{W_{2}}{\rho_{p}}}$$

where $\rho_{w}$ is the water density; *W*_1_ and *W*_2_ are the mass (g) of the water-soaked and dry hollow fiber membrane, respectively; and $\rho_{p}$ is the polymer density in (g cm^−3^).

The mean pore radius (*r_m_*) was calculated with Equation (11) [30,31].

(11) $$r_{m}=\sqrt{\frac{\left(2.9-1.75\varepsilon \right)8\eta lQ}{\varepsilon AP}}$$

where *η* is water viscosity (8.9 × 10^−9^ Pa s); *l* is the membrane thickness (m); *A* is the membrane surface area (m^2^); *P* is the operational pressure (Pa); and *Q* is the volumetric flow rate (m^3^ s^−1^).

Water contact angle: Surface wettability was assessed via static contact angle measurements using a Ramé–Hart 590 goniometer (Ledgewood, NJ, USA). For each measurement, 2 μL droplets of distilled water were placed on the surface of dense membrane samples.

## 3. Results and Discussion

### 3.1. HFM Morphology

**Figure 2:**
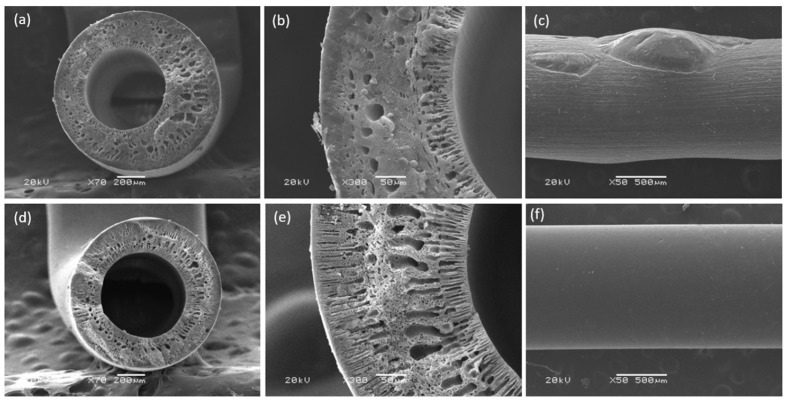
Cross-section SEM images of (**a**,**b**) 80/20 and (**d**,**e**) 80/20s with inset at ×70 and ×300. Plane-view images of (**c**) 80/20 and (**f**) 80/20s at ×50.

Initial experiments for preparing HFMs were conducted using a PPSU blend containing 20 wt% EPS waste. Figure 2 presents the cross-sectional (a, b) and plane view (c) SEM images of a PPSU/EPS blend membrane (80/20). As can be seen, the 80/20 membrane exhibits a well-defined internal and external circumference, with an EPS domain droplet morphology that is dispersed in the matrix [32,33]. The accumulation of domains within the membrane led to the formation of large-sized lumps (images C and D), which were also noticeable when handling the HFM during module preparation. This polymer segregation is attributed to the immiscibility of PPSU and EPS, where the minority phase (EPS waste) is encapsulated within the continuous phase (PPSU) [34].

**Figure 3:**
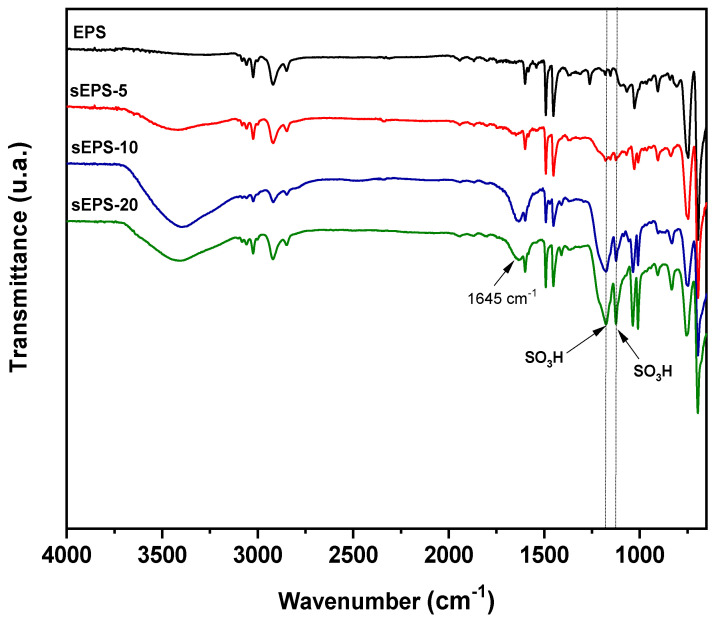
Infrared spectra of sEPS membranes at different degrees of sulfonation.

EPS waste sulfonation offers an alternative method for enhancing its compatibility with the PPSU matrix. To confirm the success of the sulfonation process with TMSClS in DCE, FTIR spectra of EPS and sEPS at different SD (5, 10, and 20%) were analyzed (see Figure 3). The characteristic bands of polystyrene were identified, including C–H stretching vibrations between 2846 and 3022 cm^−1^, aromatic C=C stretching at 1450 and 1493 cm^−1^, and a strong absorption band attributed to benzene ring C–H out-of-plane bending at 748 cm^−1^. In sulfonated EPS, two additional peaks appeared at 1127 cm^−1^ and 1181 cm^−1^, corresponding to –SO_3_H symmetric and asymmetric stretching vibrations, whose intensity increased with higher SD. In addition, a broad absorption band between 3200 and 3600 cm^−1^ due to O–H stretching vibration was observed in the sEPS samples, indicating the increased polymer hydrophilicity as SD increased. Moreover, a broad band centered at 1645 cm^−1^ was also observed in sulfonated EPS that increases with increasing SD. This feature has been attributed either to water molecules strongly associated with –SO_3_H groups or to distortions in the benzene finger region caused by disubstituted aromatic rings with the –SO_3_H group in the para-position [35].

**Figure 4:**
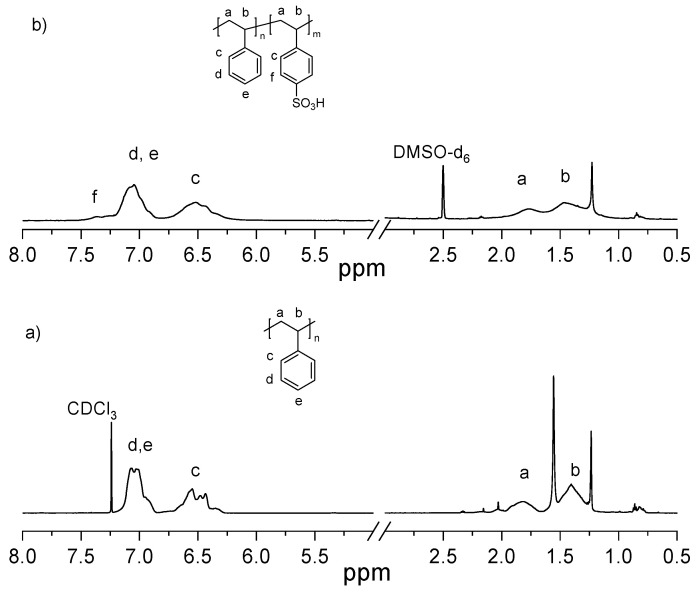
^1^H-NMR spectra of (**a**) EPS and (**b**) 5% sEPS membranes.

NMR spectroscopy also confirmed the successful sulfonation of EPS, as shown in Figure 4, where the characteristic proton signasls of both EPS and sEPS are observed, consistent with previously reported information [35]. Moreover, titration analysis further confirmed that the experimentally determined SD closely aligned with the theoretical values. A 5% SD was chosen for membrane fabrication, as higher degrees (≥10%) induce significant changes in the swelling behavior and solubility of sEPS, which complicates the formation of well-structured membranes.

**Figure 5:**
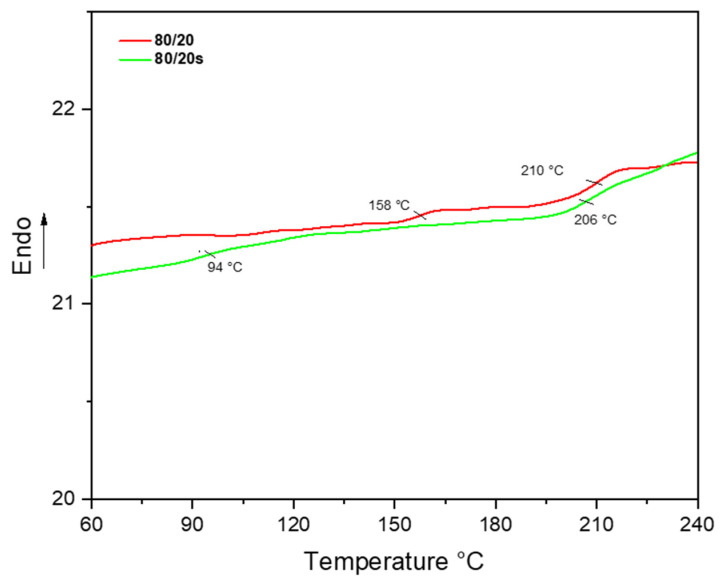
DSC curves of 80/20 and 80/20s HFMs.

**Figure 6:**
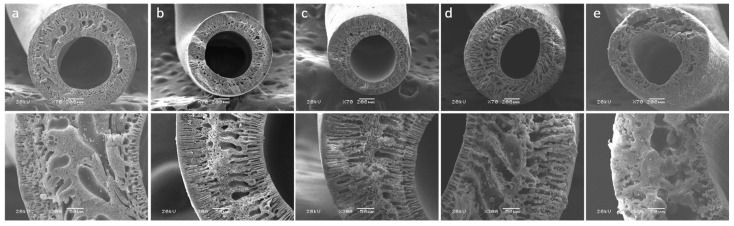
Cross-sectional SEM images of PPSU/sEPS hollow fiber membranes. Insets show higher magnification views at ×70 (top) and ×300 (bottom). (**a**) 90/10s, (**b**) 80/20s, (**c**) 70/30s, (**d**) 60/40s, and (**e**) 50/50s.

SEM images of the 80/20s membrane reveal a well-defined membrane cross-section, as shown in Figure 6e,f, with no signals of polymer segregation or large-sized lumps as seen in the 80/20 blend (Figure 2c). Specific interactions, such as inter- and intra-molecular hydrogen bonding, may occur between the –SO_3_H groups and sulfone (-SO_2_-) linkages present in PPSU, promoting an improved molecular connection between the polymers [36]. Differential scanning calorimetry (DSC) analyses corroborated these observations by revealing distinct thermal transitions associated with phase behavior (Figure 5). The 80/20 HFM exhibits two glass transition temperatures (T_g_) at 210 °C and 158 °C, which could be attributed to a pure PPSU-rich phase and a PPSU/EPS saturated blend phase, respectively. In contrast, the 80/20s membrane displayed two T_g_ values at 206 °C and 94 °C, slightly deviating from those of individual components. The shift of these temperatures towards each other indicates improved compatibility of the blend components [37]. This interpretation is consistent with the images observed in SEM of 80/20 and 80/20s HFMs. Complementary TGA results (Figure S1) show a slight weight loss (~2%) between 100 and 200 °C for the 80/20s membrane, absent in the 80/20 membrane, attributable to the release of strongly bound water associated with –SO_3_H groups. Both membranes exhibited two major degradation steps: the first between 375 and 505 °C (polymer backbone decomposition) and the second between 520 and 636 °C (carbonization processes). In both main steps, the cumulative mass loss was higher for 80/20s than for 80/20, consistent with the presence of sulfonic functionalities that promote additional volatilization, such as SO_2_ release, leading to a lower char yield at high temperature. Overall, sulfonation does not markedly alter the qualitative degradation pathway but results in slightly increased mass loss and reduced residue.

Due to the enhanced miscibility of sEPS and PPSU, it is possible to increase the EPS content in the polymer matrix, leading to benefits such as cost savings, sustainability, and improved material properties. Therefore, the effect of sEPS waste content on HFM morphology was analyzed at concentrations ranging from 10 to 50 wt%. Figure 6 shows SEM images of the PPSU/sEPS HFM cross-section. All the membranes exhibited an asymmetric cross-section morphology characterized by three visible layers, finger-like structures near the inner and outer surfaces, and a middle sponge-like structure region. The 90/10s membrane exhibited the largest macrovoids, while the 80/20s and 70/30s membranes showed a more balanced structure with well-defined layers and reduced macrovoid size. In 60/40s, the finger-like structures became more pronounced and extended, reducing the thickness of the central sponge-like region and accentuating the phase segregation. When sulfonation reached 50%, the three-layered structure disappeared entirely, giving rise to a more uniform sponge-like morphology with the largest macrovoids among all blends. These structural differences could be critical in determining the final application performance, as asymmetric membranes with macrovoids often offer increased permeability but can suffer from reduced mechanical strength or stability under certain conditions [38]. Compared to the 80/20 non-sulfonated membrane, PPSU/sEPS membranes exhibited a more refined and dispersed sEPS domain distribution, which in turn indicates that EPS sulfonation effectively improves compatibility with PPSU [20], particularly for 90/10s, 80/20s, and 70/30s blends (Figure 6a–c). However, as can be seen in Figure 6d,e, the presence of separated domains becomes evident when sEPS concentration is above 30% in the blend, with larger domain size and large porous occurrence increasing across the polymeric matrix at 40% and 50% sEPS concentrations.

Changes in the HFMs can be attributed to modifications in the thermodynamic phase inversion equilibrium during the PPSU/sEPS membrane formation process. Polymer sulfonation increases their hydrophilicity, leading to larger demixing gaps during phase inversion with water [39]. This requires larger amounts of water to induce phase separation, reducing the strength of water as a nonsolvent for sulfonated polymers [40]. This behavior helps explain the deformed inner circumference observed in the 60/40s and 50/50s membranes.

### 3.2. Solubility Parameter and PPSU/sEPS Miscibility

HSPs are widely recognized for their utility in predicting polymer miscibility and interactions with solvents [41]. The HSP system is based on *δ_d_*, *δ_p_*, and *δ_h_*, which together describe the solubility behavior of a substance in a given environment. However, HSP values are not always available for each polymer, particularly for less common or newly developed materials. Thus, HSP values, polymer–polymer miscibility (*δ_v_*), and distance *R_a_*_(*v*)_ of PPSU, EPS, and sEPS were estimated to confirm that EPS sulfonation enhances its miscibility with PPSU. These results highlight how specific molecular characteristics influence polymer compatibility (Table 3). According to Bradley’s method, miscibility between two polymers occurs when the *R_a_*_(*v*)_ parameter is ≤ 5.6 MPa^1^/^2^ [27].

In this case, sEPS exhibited a smaller *R_a_*_(*v*)_ value (4.7) compared to EPS (10.7), indicating an improved miscibility with PPSU. The improved miscibility of sEPS can be attributed to its higher *δ_p_* and *δ_h_* interactions. The presence of –SO_3_H in sEPS enhances its polarity and hydrogen capability compared to EPS, which significantly increases the polymer’s ability to form hydrogen bonds, contributing to stronger interactions with PPSU and thus improving their miscibility.

### 3.3. Dynamical Viscosity

**Figure 7:**
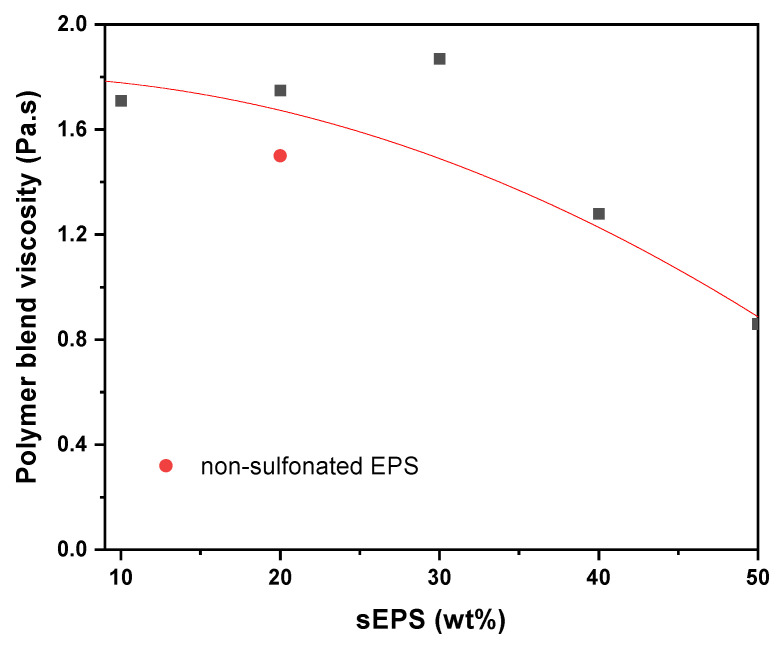
Dynamic viscosity values of PPSU/sEPS as a function of sEPS content and 80/20.

In order to better understand the relationship between the increase in sEPS content and membrane morphology, Table 1 and Figure 7 present the dynamic viscosity values as a function of the sEPS percentage in the blend. As shown in Figure 7, the introduction of sulfonated sEPS modifies polymer solution characteristics, which results in a lower flow resistance as sEPS content increases. However, a deviation was observed for the 70/30s blend, which exhibited a higher viscosity compared to pure PPSU and the other PPSU/sEPS blends. The viscosity behavior observed in the 70/30s solution followed similar trends to those reported by Lin for polystyrene (PS)/polybutylene (PB) blends [42]. Viscosity initially decreased as the PS amount increased, but at around 30% PS, a sudden viscosity increase was observed, followed by a further decrease as PS content continued to rise. Lin attributed this unusual viscosity behavior to a discontinuity in velocity and stress at the interface between the two polymers, a phenomenon known as interlayer slip. Okoroafor et al. reported that interlayer slip occurs when there is a dilation at the interface between two immiscible polymer compositions [43]. The resulting phase separation at the interface can cause an increase in viscosity, as the system flow is hindered by the differing dynamics between the two polymer components. In this case, the increase occurs at 30% sEPS concentration, where the PPSU/sEPS blend reaches a critical point in terms of phase compatibility. On the other hand, PPSU/sEPS’s higher viscosity with up to 30% sEPS compared to those containing 20% non-sulfonated EPS can be attributed to the formation of hydrogen bonding and steric hindrance between sEPS –SO_3_H groups and PPSU chains, which significantly improves blend compatibility [44].

### 3.4. Mean Pore Size and Porosity

**Figure 8:**
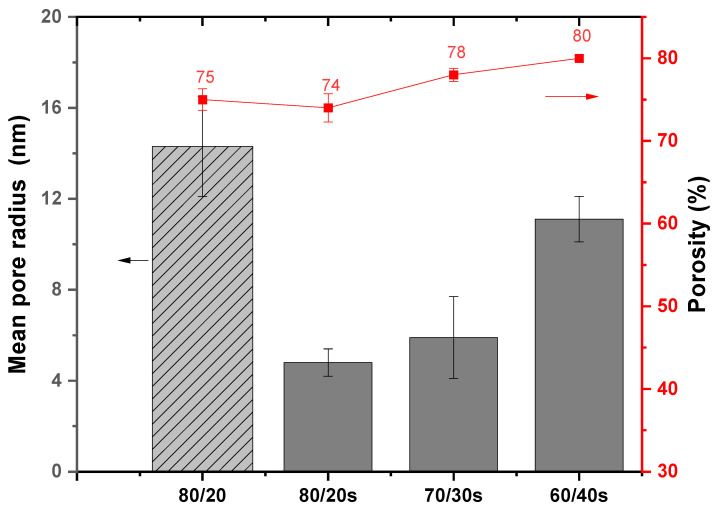
Mean pore radius and percentage porosity of 80/20, 80/20s, 70/30s, and 60/40s HFMs.

The results of mean pore radius and porosity are presented in Figure 8. The following trend in pore radius for PPSU/sEPS HFM was observed: 80/20s (4.8 ± 0.6) < 70/30s (5.9 ± 1.8) < 60/40s (11.1 ± 1.0). However, a significant increase in mean pore radius is observed in the 80/20 membrane containing non-sulfonated EPS (14.3 ± 2.2 nm) and in 60/40s (11.1 ± 1.0 nm). The tested membranes presented porosity values between 74 and 80%. Despite the similarity in porosity observed in all membranes, a poor interaction between polymers in the PPSU/EPS 80/20 becomes apparent through the presence of large agglomerates and larger droplets. The 90/10s and 50/50s were excluded due to their low and high sEPS content, respectively.

### 3.5. Mechanical Properties

Compared to flat-sheet membranes, HFMs offer a higher specific surface area, and they are mechanically self-supporting, allowing for easy assembly into modules [45]. However, they are more prone to damage or breakage under high pressure. The loss of mechanical strength can result in HFM instability, the formation of surface cracks, and disruption in the liquid or gas separation process, among other issues [46]. In this context, the mechanical properties of the PPSU/sEPS HFM membranes were evaluated.

**Figure 9:**
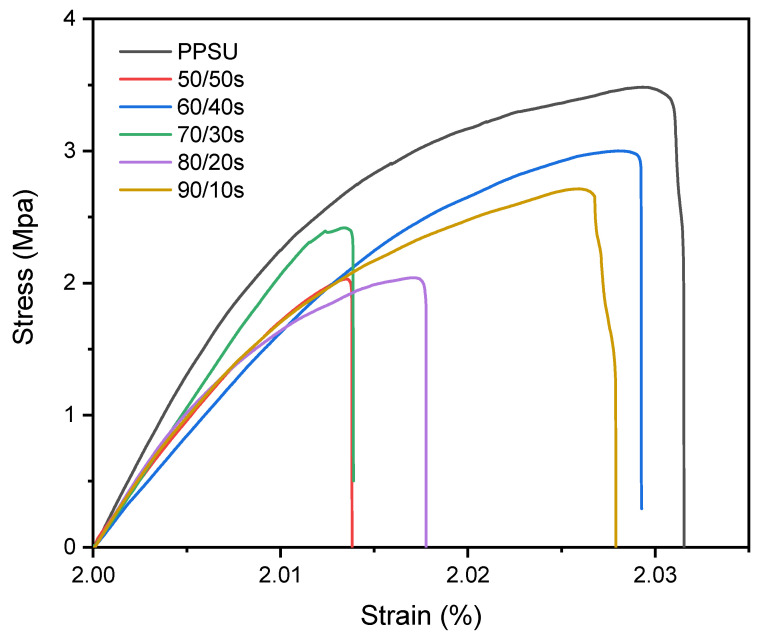
Stress–strain curves of PPSU/sEPS hollow fiber membranes.

As shown in Table 4 and Figure 9, PPSU/sEPS HFM exhibited a decrease in both elastic modulus (E) and tensile strength (TS) with increased sEPS waste content compared to pure PPSU. The result is attributed to the incorporation of brittle EPS in the PPSU matrix and the increase in free volume caused by the presence of bulky –SO_3_H groups.

In contrast, an increase in both E and TS was observed in PPSU/sEPS membranes in comparison with the 80/20 membrane containing non-sulfonated EPS, suggesting better miscibility between sEPS and PPSU. Improved miscibility is attributed to intermolecular interactions, which contribute to a more rigid structure and better overall mechanical properties [47]. Elongation at break (ε) values remained almost the same when compared to pure PPSU. Morphological parameters, such as pore size and finger-like macropores, could also be affecting mechanical properties of PPSU/sEPS membranes [48]. Despite the reduction in mechanical properties in comparison with pure PPSU membrane, PPSU/sEPS waste membranes meet the requirements for nanofiltration applications.

### 3.6. Permeability and Flux Recovery Ratio (FRR %)

As shown in Table 5, HFM water flux (J_w1_) at 2 bar increased from 4.7 to 21.2 L m^−2^ h^−1^ as the sEPS content in the blend increased from 20 to 40%. This trend is consistent with pore size measurements, where membranes with smaller mean pore radii, 80/20s (4.8 ± 0.6) and 70/30s (5.9 ± 1.8), exhibited lower fluxes, while those with larger pore sizes, 60/40s (11.1 ± 1.0) and 80/20 (14.3 ± 2.2), achieved higher fluxes. Notably, the enhanced permeability of the 80/20 membrane is attributed to microfissures resulting from poor polymer compatibility.

Regarding antifouling performance, the FRR% revealed distinct trends depending on sEPS content and foulant type. For BSA, the non-sulfonated 80/20 HFM exhibited poor fouling resistance with an FRR of 31.0 ± 8.6%. In contrast, PPSU/sEPS membranes showed fairly constant FRR values with increasing EPS content: 79.9 ± 4.9% for 80/20s, 78.1 ± 0.5% for 70/30s, and 80.9 ± 6.0% for 60/40s, demonstrating that sulfonation improves antifouling capacity against BSA. For xanthan gum (XG), a larger molecule, FRR shows a dependence on sulfonation. The 80/20 non-sulfonated membrane exhibited a lower FRR (69.8 ± 9.5%), increasing to 91.9 ± 1.1% for 80/20s and peaking at 99.3 ± 0.2% for 70/30s, before declining to 74.8 ± 16% at 60/40s. This decrease is attributed to the larger domain sizes and increased porosity at higher sEPS content (40%), which may facilitate gel-layer formation or deeper foulant penetration, reducing antifouling efficiency. The higher variability observed in the FRR of the 60/40s membrane (±16%) can also be explained by the structural heterogeneity seen in SEM (Figure 6d,e) and the significant increase in mean pore radius (11.1 ± 1.0 nm), which leads to less uniform fouling behavior among replicate fibers.

**Figure 10:**
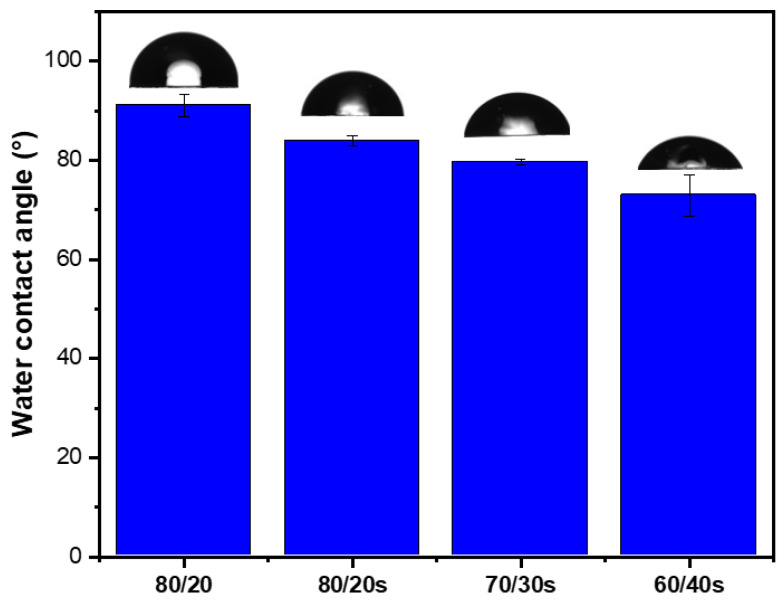
Water contact angle values of membranes. Representative droplet images are shown above each bar.

Since increased hydrophilicity is generally associated with improved antifouling performance, water contact angle measurements were conducted (Figure 10). A decrease in hydrophobicity with increasing sEPS content in the membranes was observed. The 80/20 membrane exhibited a contact angle of 91.1° ± 2.3°, indicating a hydrophobic surface, which is consistent with the inherent hydrophobic nature of EPS. However, incorporation of sEPS led to a progressive reduction in contact angle: 80/20s (83.9° ± 1.1°), 70/30s (79.6° ± 0.6°), and 60/40s (72.9° ± 4.2°), indicating enhanced surface hydrophilicity. This trend is consistent with water uptake data (Table S1, Supplementary Material), where sulfonated membranes exhibited values close to 1% compared to only 0.28% for the non-sulfonated 80/20 membrane. Weight loss remained below 1% in all cases, confirming that the membranes maintained good structural stability in aqueous environments.

Moreover, the permeability and FRR% of 80/20s and 70/30s were measured at 3 bar. Water fluxes increased under higher pressure, in particular for the 70/30s membrane. However, FFR values for both BSA and XG decreased, consistent with the trend observed at 2 bar. As pressure increases, fouling becomes more severe due to the enhanced compression of foulants onto the membrane surface, making their removal more difficult during cleaning [49].

### 3.7. Membrane Dye Separation Performance

**Figure 11:**
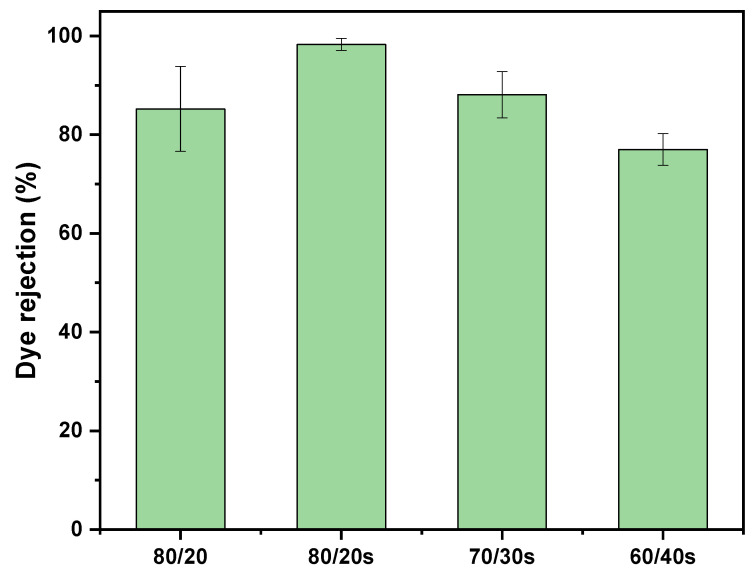
Reactive Black 5 rejection of 80/20, 80/20s, 70/30s, and 60/40s membranes at 2 bar pressure.

The separation performance of the PPSU/sEPS membranes was evaluated using RB5 (Mw: 991.82 g/mol). Membrane dye rejection (Figure 11) followed the order 80/20s > 70/30s > 80/20 > 60/40s, with most membranes showing a trend consistent with pore size, except for 60/40s, which showed a lower rejection than 80/20. Higher rejection performance in membranes with smaller pore sizes is consistent with steric hindrance mechanisms. In addition, the negatively charged nature of the PPSU/sEPS membranes, due to the deprotonation of the sulfonic groups, may further enhance selectivity through electrostatic interactions [50]. A negative surface potential of the membranes can be expected, which, according to the Gouy–Chapman theory, would form an electric double layer that repels anionic solutes such as RB5, thereby increasing their rejection [51]. This is consistent with literature reports indicating that PPSU membranes exhibit a negative surface potential around pH 7, which becomes more negative upon sulfonation [29], and that polystyrene shows a negative zeta potential of approximately –60 mV at pH 7 [52]. Together, these observations suggest that electrostatic repulsion contributes to the enhanced rejection of anionic solutes such as RB5.

**Figure 12:**
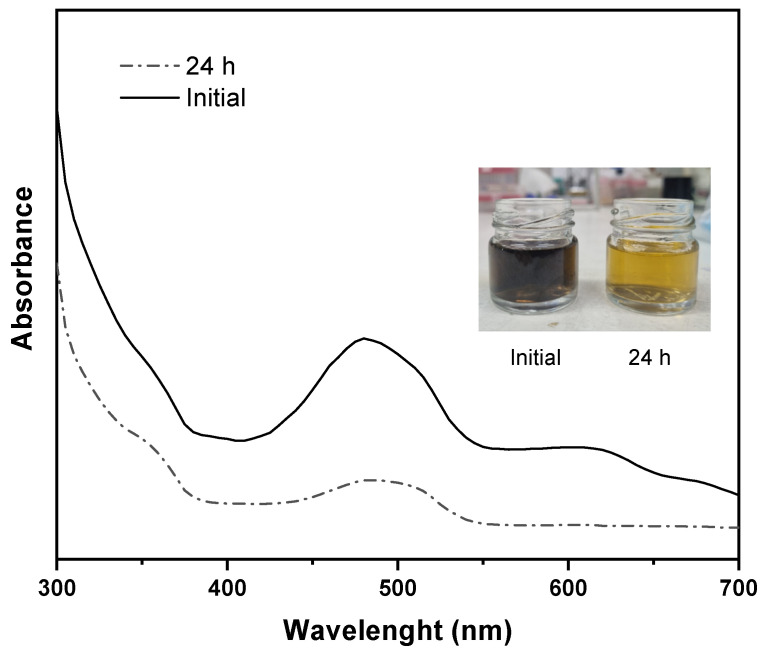
Dye mixture absorbance spectra before and after passing through an 80/20s membrane.

A dye mixture containing MO, RB5, CR, and MB was tested to assess dye separation under more realistic conditions [53]. The significant spectral overlap of these dyes makes them difficult to separate using traditional methods [54]. In this study, we used the standard UV–vis method to measure the overall color intensity of a dye mixture at a concentration of 50 ppm, rather than determining the individual concentrations of each dye. As depicted in Figure 12, mixing the four dyes resulted in a new color, with the UV–vis spectra showing a single peak.

**Figure 13:**
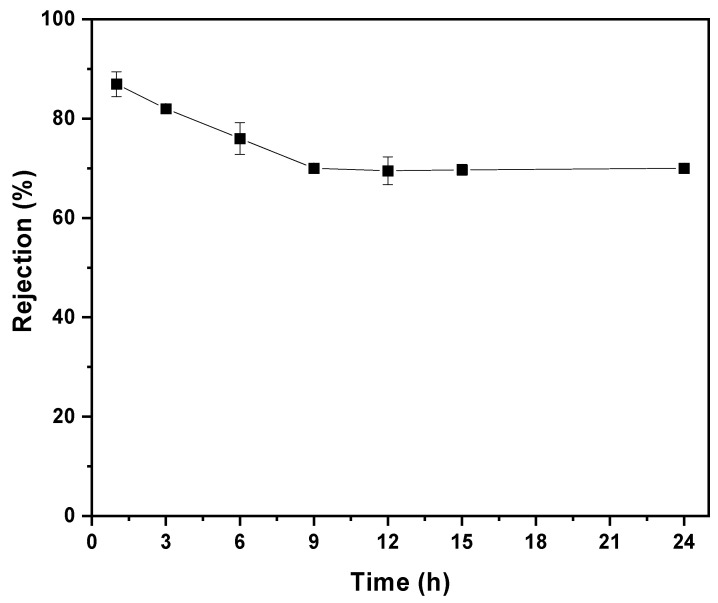
Dye mixture separation through 80/20s membrane during a 24 h test.

The 80/20s membrane was chosen for the dye mixture’s rejection due to its favorable separation and antifouling performance. During a 24 h continuous test at 2 bar, the overall rejection ranged from 86.1% to 70.3% (Figure 13). Dye rejection stabilized at 70% after 9 h as equilibrium was reached. The observed decrease in rejection can be attributed to the reduced volume (~ 8%) of the dye–water mixture that permeated during the test, as the rejection performance of charged membrane is known to be influenced by the solute concentration in the remaining solution. Moreover, the permeate flux remained around 4.3 L m^−2^ h^−1^ throughout the experiment, indicating consistent filtration performance under the tested conditions.

Table 6 presents PPSU/sEPS membranes’ RB5 rejection compared to other reported hollow fiber NF membranes. Among the tested membranes, the 80/20s membrane exhibited superior separation properties compared to the 70/30s and 60/40s membranes. Although the 80/20s membrane showed slightly lower dye rejection and permeability values than the POMs-M membrane (containing polyoxometalates functionalized on alumina/PEI hollow fibers), it offers the advantage of incorporating a percentage of recycled polymer. This characteristic enhances the sustainability of the membrane by promoting waste reduction and the principles of the circular economy without significantly compromising its functional performance. When compared with PAN(92)-co-P2EHA(8), the 80/20s and 70/30s membranes demonstrated higher RB5 rejection. However, it is important to note that the performance test for PAN(92)-co-P2EHA(8) was conducted at a higher dye concentration, which could partially explain the observed differences. Moreover, the 80/20s membrane showed slightly higher dye rejection than the PES [55], PPSU/ZSM-5 [56], and HF 0.30 membranes [57], indicating its competitive performance for dye rejection applications. Meanwhile, the 70/30s membrane exhibited dye rejection performance comparable to those of chitosan-based nanoparticle/PPSU membranes [57], although it showed lower permeability. This suggests a trade-off between rejection efficiency and membrane permeability, which should be considered for practical applications. PZ-0 HFM (containing PPSU and 2% PVP) achieved less than 58% RB5 rejection at 4 bar using a 100 ppm solution, while flat-sheet PSF membranes reported under 60% rejection at 25 bar with a 40 ppm RB5 solution [56,58]. In contrast, the 80/20s membrane achieved ~98% rejection at only 2 bar, highlighting its effective separation performance at lower pressure.

Previous studies have explored a broad range of RB5 concentrations, where higher concentrations are generally associated with lower rejection rates. The results obtained in this study are comparable to those reported at concentrations of 50, 100, and 20 ppm. However, it is important to highlight that this study incorporates recycled polymer into the membrane. This included innovation offers a dual benefit: maintaining effective RB5 rejection performance while contributing to environmental sustainability through the use of recycled waste polymers.

### 3.8. Energy Consumption and Sustainability of the sEPS Process

It is important to consider energy consumption and required reagents for the sulfonation process, factors that significantly affect both economic feasibility and environmental impact. The detailed methodology used for the Aspen simulation is provided in the Supplementary Material (Section 1). EPS sulfonation involves the use of reagents such as the sulfonation agent TMSClS and solvents DCE and EtOH, which, despite being widely used in industrial processes, present environmental concerns due to their potential toxicity and contribution to volatile organic compound emissions. In terms of energy consumption, the Aspen simulation results indicate that the total energy consumption of the process is 53.9 Wh per kg of sEPS, which includes 45 Wh attributed to the pumps and 8.77 Wh for the drying process (equivalent to 0.00194 kg CH_4_/kg). This energy demand is relatively low when compared to high-performance thermoplastic production such as polysulfone (PSU), PPSU, polycarbonate (PC), or PET, which requires between 13.8 and 50 kW/kg, depending on the process conditions treatment [62,63,64].

Moreover, it is important to highlight that the EPS sulfonation process could benefit from further optimization. For example, incorporating a distillation column at the exit of the precipitation tank (see Figure S2) to recover and recycle solvents could significantly enhance process efficiency and ensure proper management of solvents and waste generation. Additionally, replacing fossil fuels with an electric furnace powered by renewable energy could improve the sustainability of the process. However, exploring these improvements lies beyond the scope of the current work. Overall, the incorporation of 20 or 30% of recycled sEPS into PPSU-based HFMs offers a significant reduction in PPSU consumption, which leads to lower energy costs and a decrease in the environmental impact of membrane production, without compromising material properties.

Future work should include long-term operation tests (72–168 h) and chemical stability evaluations under different pH (3–11) and temperature (25–45 °C) conditions, as well as studies with more realistic industrial wastewater containing salts, surfactants, and other organic contaminants to validate membrane durability under realistic scenarios. In addition, comprehensive pressure stability tests are needed to confirm structural integrity under higher operational stresses, and full life cycle and cost analyses will be essential to establish the overall environmental and economic viability of the process.

## 4. Conclusions

Asymmetric hollow fiber membranes were successfully prepared from PPSU with varying concentrations of recycled sEPS via the phase inversion process, demonstrating promising antifouling and separation properties. The introduction of –SO_3_H groups into sEPS improved miscibility with PPSU, enhanced hydrophilicity, and imparted antifouling characteristics, while maintaining the structural and mechanical integrity of the membranes.

Sulfonation significantly improved surface hydrophilicity, with the contact angle decreasing from 91.1 for 80/20 to 72.9 for the 60/40s membrane, confirming enhanced water affinity at the surface. In terms of mechanical behavior, PPSU/sEPS membranes exhibited lower tensile strength and elastic modulus compared to pure PPSU, as expected due to EPS incorporation, but retained sufficient robustness for their application. The 80/20s HFM achieved 98.3% rejection of RB% and maintained ~70% rejection in a 24 h mixed dye test, while the 70/30s membrane reached a flux recovery ratio of 99.3% against XG solution, confirming its excellent antifouling capacity.

Overall, PPSU/sEPS blend membranes offer dual advantages: high-performance separation capabilities and enhanced environmental sustainability through the incorporation of recycled polymer. These membranes emerge as a promising and sustainable option for diverse industrial separation applications.

## Figures and Tables

**Table 1:** Amounts of polymers in each dope solution and their dynamic viscosities.

HFM	PPSU (g)	sEPS (g)	Viscosity (Pa.s)
90/10s	4.5	0.5	1.71 ± 0.08
80/20s	4.0	1.0	1.69 ± 0.05
80/20 *	4.0	1.0	1.50 ± 0.04
70/30s	3.5	1.5	1.87 ± 0.05
60/40s	3.0	2.0	1.28 ± 0.10
50/50s	2.5	2.5	0.86 ± 0.09

* Polymeric blend of PPSU with 20% non-sulfonated EPS.

**Table 2:** Spinning parameters for the elaboration of PPSU/sEPS HFMs.

Spinning Parameters	Operating Conditions
Dope flow rate (mL/min)	2.8
Bore fluid flow rate (mL/min)	0.8
Bore fluid	Deionized water
External coagulant	Tap water
External coagulant temperature	23 °C
Dope temperature	23 °C
Air gap distance (cm)	3.5
Concentration of dope solution (%)	18
Spinneret outer/inner diameter of needle (mm)	1.6/0.337

**Table 3:** Calculated HSPs, calculated *δ_v_*, and distance *R_a(v)_* of PPSU with EPS or sEPS.

Polymer	$\delta_{d}$	$\delta_{p}$	$\delta_{h}$	*δ_v_*	*R_a_*_(*v*)_ (MPa^1/2^)
EPS	20.2	1.2	0	1.2	10.7
sEPS	20.1	3.8	4.2	5.7	4.7
PPSU	18.7	5	7.4	9.0	-

**Table 4:** Mechanical properties of 80/20 HFM and from PPSU/sEPS with different sEPS content.

HFM	E (MPa)	TS (MPa)	ε (%)
PPSU	547.3 ± 38.96	4.04 ± 0.67	2.04 ± 0.01
90/10s	243.7 ± 13.26	3.19 ± 0.53	2.03 ± 0.01
80/20 *	183.0 ± 5.67	2.52 ± 0.18	2.04 ± 0.01
80/20s	241.2 ± 11.64	2.98 ± 0.71	2.05 ± 0.02
70/30s	235.9 ± 21.87	2.96 ± 0.99	2.04 ± 0.02
60/40s	234.5 ± 27.24	3.66 ± 1.27	2.02 ± 0.01
50/50s	207.2 ± 23.83	3.34 ± 0.42	2.03 ± 0.01

* Polymeric blend of PPSU with 20% non-sulfonated EPS.

**Table 5:** Water flux and FRR values and water contact angle of PPSU, 80/20, 80/20s, 70/30s, and 60/40s.

Membrane			BSA Solution		XG Solution	
	Water Flux J_w1_ (L m^−2^ h^−1^)		Flux Recovery Ratio (FRR%)		Flux Recovery Ratio (FRR%)	
Pressure (bar)	2	3	2	3	2	3
80/20	31.0 ± 8.6	-	47.2 ± 5.6	-	69.8 ± 9.5	-
80/20s	4.7 ± 1.2	6.9 ± 0.8	79.9 ± 4.9	80.7 ± 2.0	91.9 ± 1.1	66.8 ± 2.7
70/30s	6.4 ± 2.9	24.1 ± 7.1	78.1 ± 0.5	64.2 ± 6.7	99.3 ± 0.2	76.1 ± 13
60/40s	21.2 ± 3.4	-	80.9 ± 6.0	-	74.8 ± 16	-

**Table 6:** Separation performance and pure water permeation (PWP) of some reported NF hollow fiber membranes in comparison with membranes reported in this work.

Membrane	Dye Concentration (ppm)	RB5 Rejection (%)	PWP (L m^−2^ h^−1^)	P (Bar)	Ref.
POMs-M	20	100	19	0.5	[59]
Polysulfone-polyamide	500–2000	60–97	0.0059–0.0206	1.7	[60]
PAN(92)-co-P2EHA(8)	100	45.1	85.8	2	[61]
PES	50	96.7	66	5	[55]
PPSU/ZSM-5	100	90.8	113.9	3	[56]
HF 0.30	10	89.2	96.8	2	[57]
PZ-0 (PPSU/2%PVP)	100	58	26.2	4	[58]
PSF	40	<60	26.8	25	[56]
80/20s	50	98.3	4.7	2	This work
70/30s	50	88.1	6.4	2	This work
60/40s	50	77.0	21.2	2	This work

## Data Availability

The original contributions presented in this study are included within this article; further inquiries can be directed to the corresponding author.

## Supplementary Materials

The following supporting information can be downloaded at: https://www.mdpi.com/article/10.3390/membranes15100285/s1, Figure S1: Thermogravimetric (TGA) and derivative thermogravimetric (DTG) curves of 80/20 and 80/20s membranes; Figure S2: Batch sulfonation of EPS with TMSClS at room temperature; Table S1: PPSU/sEPS HFMs water uptake and weight loss characteristics.
