# Supplementary material for: Recycled Polystyrene as a Sustainable Material for Hollow Fiber Membranes in Dye Filtration
Source: Membranes (Basel). 2025 Sep 23;15(10):285. doi: 10.3390/membranes15100285 (PMC12566264; doi:10.3390/membranes15100285)
Supplement: Supplementary file 1 [file membranes-15-00285-s001.zip › membranes-3856662-supplementary.pdf]

Recycled Polystyrene as a Sustainable Material for Hollow Fiber Membranes in Dye Filtration

Mauricio Huhn-Ibarra1, Libia Madai Itza-Uitzil1,2, Marcial Yam-Cervantes1, Abigail González-Díaz3, Fernando José Zapata-Catzin1, Javier Ivan Cauich-Cupul1, Manuel Aguilar-Vega1, Maria Ortencia González-Díaz4*

1 Centro de Investigación Científica de Yucatán, A.C., Calle 43 No. 130, Chuburná de Hidalgo, C.P. 97200, Mérida Yucatán, México.
2 Tecnológico Nacional de México, Instituto Tecnológico de Mérida, Av. Tecnológico S/N Km. 4.5 C.P. 97118, Mérida Yuc. México.
3 El Colegio de Puebla A.C., Av. 141 pte. 505, Gabriel Pastor 1ra. Sección, Puebla, C.P. 72420, México.
4 *SECIHTI - Centro de Investigación Científica de Yucatán, A.C., Calle 43 No. 130, Chuburná de Hidalgo, 97200, Mérida Yucatán, México.*
* Correspondence: maria.gonzalez@cicy.mx

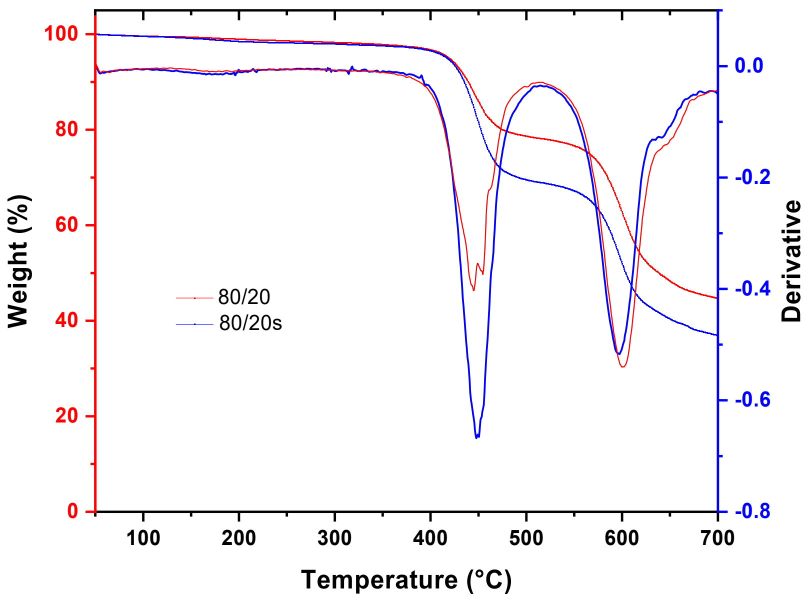

Figure S1. Thermogravimetric (TGA) and derivative thermogravimetric (DTG) curves of **80/20** and **80/20s** membranes.

1.- Simulation of energy consumption in the EPS sulfonation process

EPS sulfonation process, as shown in Figure S1 (dotted section in the process), was simulated in ASPEN Plus V12 to estimate electrical and thermal energy consumption. The simulation assumes a sEPS production capacity of 1 kg h^{-1}, with 100% conversion and a 97% yield. In addition, the operating conditions are assumed to be the same as those in the experiments. The main electricity consumes are four pumps: two pumps supply DCE and TMSCIS to the batch reactor, respectively, the third pump transfers the reactor products to the precipitation tank, and the fourth pump moves EtOH from

the storage tank to the precipitation tank. The main thermal energy consumer is the dryer, which heats the sEPS from 23.8 to 70 °C to remove residual liquids. Natural gas is used in the dryer to heat the air to 70 °C, the air then provides the heat necessary to dry sEPS.

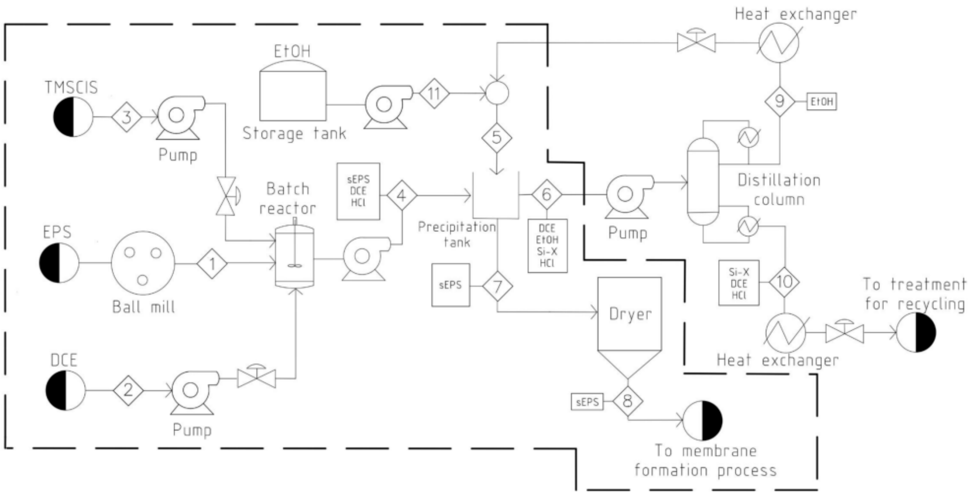

Figure S2. Batch sulfonation of EPS with TMSClS at room temperature.

Table S1. PPSU/sEPS HFMs water uptake and weight loss characteristics

**HFMs**	**Water uptake** %	**Weight loss** %
**90/10s**	1.19 ± 0.32	0.65 ± 0.35
**80/20s**	1.11 ± 0.06	0.51 ± 0.19
**80/20***	0.28 ± 0.08	0.40 ± 0.55
**70/30s**	1.12 ± 0.05	0.42 ± 0.59
**60/40s**	1.05 ± 0.19	0.92 ± 0.19
**50/50s**	1.05 ± 0.02	0.45 ± 0.63

*Polymeric blend of PPSU with 20% non-sulfonated EPS
